# Effect of Eradication of *Helicobacter pylori* on Expression Levels of FHIT, IL-8 and P73 in Gastric Mucosa of First-Degree Relatives of Gastric Cancer Patients

**DOI:** 10.1371/journal.pone.0124576

**Published:** 2015-04-14

**Authors:** Juan Liao, Shichao Wen, Lipeng Cao, Yunfeng Zhou, Zhisong Feng

**Affiliations:** Department of Gastroenterology, The Affiliated Hospital of North Sichuan Medical College, 63 Wenhua Road, Nanchong 637000, Sichuan Province, China; Instituto de Tecnologia Quimica e Biologica, PORTUGAL

## Abstract

**Objectives:**

*Helicobacter pylori* (*H*. *pylori*) infection plays an important role in the carcinogenesis and development of gastric cancer. Eradication of *H*. *pylori* can effectively reduce the risk of gastric cancer, but the underlying mechanisms are not fully understood. This study aimed to investigate the effect of eradication of *H*. *pylori* on the expression levels of FHIT, IL-8 and P73 in the gastric mucosa of first-degree relatives of gastric cancer patients.

**Methods:**

One hundred and thirty-two patients with functional dyspepsia having first-degree relatives with gastric cancer were prospectively recruited in this study. Nine patients presented with *H*. *pylori* infection and family histories of gastric cancer, 61 with *H*. *pylori* infection and without family histories of gastric cancer, 6 without *H*. *pylori* infection and with family histories of gastric cancer, and 56 without *H*. *pylori* infection and family histories of gastric cancer. The protein and mRNA expression levels of FHIT, IL-8 and P73 in gastric mucosa of the subjects were detected by immunohistochemical staining and polymerase chain reaction, respectively.

**Results:**

Compared with the patients without *H*. *pylori* infection and family histories of gastric cancer, both the protein and mRNA levels of FIHT significantly decreased in patients with *H*. *pylori* infection and/or family histories of gastric cancer, and both the protein and mRNA levels of IL-8 significantly increased. After eradication of *H*. *pylori*, both the protein and mRNA levels of FHIT were significantly higher, and both the protein and mRNA levels of IL-8 were significantly lower. However, *H*. *pylori* infection and family histories of gastric cancer had no major effect on P73 expression.

**Conclusions:**

Down-regulation of FHIT and up-regulation of IL-8 may be involved in the pathogenesis of *H*. *pylori* infection in the first-degree relatives of gastric cancer patients.

## Introduction

Gastric cancer continues to be a significant worldwide problem. It is the fourth most common cancer and second leading cause of cancer mortality in the world [1]. Although the mechanisms underlying its carcinogenesis remain unclear, many environmental and genetic factors are proved to play an important role in the carcinogenesis and development of gastric cancer [2]. *Helicobacter pylori (H*. *pylori)* infection has been identified to be the most important environmental factor [2–4]. However, *H*. *pylori* infection can cause different outcomes such as atrophic gastritis, peptic ulcer, gastric cancer and gastric mucosa-associated lymphoid tissue lymphoma [5]. Epidemiologically, half of the world’s population is infected with *H*. *pylori*, but less than 2% of this population develops into gastric cancer [6]. Thus host genetic susceptibility is involved in different outcomes of *H*. *pylori* infection [7]. Family histories of gastric cancer, especially first-degree relatives of gastric cancer patients, reportedly increase the risk of developing gastric cancer [8]. Moreover, *H*. *pylori* infection and family histories of gastric cancer have synergistic effect on the carcinogenesis and development of gastric cancer [9]. Eradication of *H*. *pylori* can effectively reduce the risk of gastric cancer [10, 11], but the underlying mechanisms are not fully understood.

Gene alterations have been indentified to play a vital role in the carcinogenesis and development of gastric cancer [12, 13]. The fragile histidine triad (FHIT) gene is a tumor suppressor gene involved in various types of human cancers [14–18]. Loss of FHIT expression has been found in the gastric mucosa of patients with gastric carcinoma [19]. Stec-Michalska et al demonstrated that FHIT expression significantly decreased in the gastric mucosa of patients with functional dyspepsia and *H*. *pylori* infection [20, 21]. Moreover, FHIT expression in gastric carcinoma is related to the type, grade, and stage of the tumor and FHIT is an independent predictor for cancer-specific/overall survival of patients with gastric cancer [22, 23]. Interleukin-8 (IL-8) is a central mediator of the inflammatory response to *H*. *pylori* infection and is associated with the development of *H*. *pylori*-associated gastroduodenal disease [24]. Moreover, IL-8 is a strong stimulator of angiogenesis in gastric adenocarcinoma [25, 26]. P73 shares remarkable sequence identity to P53, especially the DNA-binding domain, suggesting that it may be another anti-oncogene. However, over-expression of P73 commonly exists in matched tumor/nontumor adjacent mucosa of patients with early gastric cancer and rarely in chronic gastritis patients [27], and wild-type P73 is frequently over-expressed in gastric carcinoma tissues [28]. In addition, interaction of *H*. *pylori* with gastric epithelial cells leads to robust up-regulation of P73 protein in vitro and in vivo in human gastritis specimens and *H*. *pylori*-infected mice [29]. However, the effects of eradication of *H*. *pylori* on the expression levels of FHIT, IL-8 and P73 remain unclear. In this study, we investigated the expression levels of FHIT, IL-8 and P73 in the gastric mucosa of patients with functional dyspepsia having first-degree relatives with gastric cancer, and also analyzed the effects of eradication of *H*. *pylori* on the expressions of FHIT, IL-8 and P73.

## Patients and Methods

### Patients

This study was approved by the Human Ethics Review Committee of North Sichuan Medical College. Written informed consent was obtained from patients in accordance with the Declaration of Helsinki and its later revision. All specimens were handled and made anonymous according to the ethical and legal standards. All patients were of the same ethnicity (Chinese).

From January 2008 to December 2013, 132 consecutive patients (81 males, 51 females; aged from 26 to 68 years) with functional dyspepsia having first-degree relatives with gastric cancer were recruited prospectively in this study. All patients underwent endoscopic examination in our hospital. The patients were assigned to four groups as follows: group A (patients with both *H*. *pylori* infection and family histories of gastric cancer), group B (patients with *H*. *pylori* infection and without family histories of gastric cancer), group C (patients with family histories of gastric cancer and without *H*. *pylori* infection), group D (patients without both *H*. *pylori* infection and family histories of gastric cancer).

Patients who were treated with *H*. *pylori* eradication therapy, antibiotics, bismuth-containing compounds, H_2_-receptor blockers or proton pump inhibitors within four weeks were excluded from this study.

Biopsies were performed before and after eradication of *H*. *pylori*. Six samples were obtained from each patient under endoscopic examination. All samples were located in the mucosa within 3–5 cm from the pylorus. Three samples were used for immunohistochemical staining and the other three samples were used for real-time quantitative reverse transcription polymerase chain reaction (qRT-PCR) assay.

### Eradication of *H*. *pylori*

For eradication of *H*. *pylori*, the patients in groups A and B were treated with esomeprazole (40 mg, bid), amoxicillin (1.0 g, bid) and clarithromycin (0.5 g, bid) for 10 days. Four weeks after drugs withdrawal, rapid urease tests were performed on those patients. If the tests showed positive results of *H*. *pylori* infection, the patients were further treated with esomeprazole (40 mg, bid), bismuth potassium citrate (240 mg, bid), levofloxacin (500 mg, qd) and furazolidone (100 mg, bid) for 10 days. Four weeks after drugs withdrawal, rapid urease tests were also performed on these patients. If the results were still positive, the patients were excluded from the study.

### Urea breath test

After fasting for more than 6 h, all patients swallowed a ^14^C-urea capsule with water. Breath samples were collected with a bottle containing CO_2_ absorbent after 10 min. All patients exhaled gently into the bottle until the CO_2_ absorbent changed in color from purple to colorless transparent. The bottle was then inserted into the sample slot of an instrument to detect the value of disintegrations per minute (DPM). A breath sample DPM<50 was defined as a negative result. DPM≥200 was defined as a positive result. DPM in the range of 50–199 was classified as indeterminate.

### Immunohistochemical staining

The staining procedures were performed as described by Zhao et al [30]. In brief, formalin-fixed paraffin-embedded sections were cut at 4 μm and placed on APES-coated slides. The slides were deparaffinized in xylene and rehydrated with graded alcohol, and 3% H_2_O_2_ was applied for 15 min at room temperature to block endogenous peroxide activity. The slides were heated in 0.01 mol/L citrate buffer (pH 6.0) in a microwave oven for 2 h at 60°C for antigen retrieval, blocked with 2% horse serum for 10 min at room temperature, and incubated with FHIT, IL-8 or P73 antibodies (1:200) in a moist chamber at 4°C overnight. The avidin-biotin-peroxidase complex procedure (ABC) was then performedusing an ABC immunohistochemistry kit. Peroxidase activity was detected with diaminobenzidine as a substrate. Finally, the sections were weakly counterstained with Harris’s haematoxylin. PBS was used as a negative control, and normal mucosa was used as a positive control.

**Fig 1:**
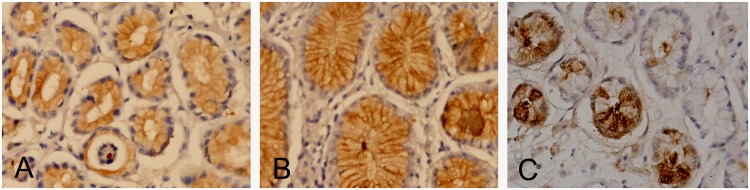
Protein expression of FHIT, IL-8 and P73 showed by immunohistochemical staining of gastric mucosa specimens from patients without both *H*. *pylori* infection and family histories of gastric cancer. A. Expression of FHIT, ×40; B. Expression of IL-8, ×40; C. Expression of P73, ×40.

Both the extent and intensity of immunopositivity were considered when scoring the protein expression levels of FHIT, IL-8 and P73. FHIT was mainly located at the cell plasma (Fig 1A), P73 at the cell nuclei (Fig 1B), and IL-8 at the cell membrane (Fig 1C). The cells containing yellow-brown granules were considered as positive cells. Five high-power fields were randomly selected in each slice, and the staining intensity and percentage of positive cells were assessed by counting 200 cells per high-power field with five sections (every fourth section) per sample. The extent of positivity was scored as follows: 0, positive cells <5%; 1, 5%-25%; 2, 25%-50%; 3, 50%-75%; and 4, >75%. The intensity was scored as follows: 0, negative; 1+, weak; 2+, moderate; and 3+, strong. The final score was obtained by multiplying the extent of positivity and intensity scores, producing a range from 0 to 12 [31, 32].

### Real-time qRT-PCR assay

For qRT-PCR experiments, dissected gastric mucosa tissues were immediately snap-frozen in liquid nitrogen. Total RNA was extracted using Trizol reagent (Life Technologies) following the manufacturer’s protocol. qRT-PCR was performed according to standard protocols. For cDNA synthesis, 2.5 μg of total RNA was reverse transcribed with Superscript II (Life Technologies) in a volume of 50 μl according to the manufacturer’s instructions. The reaction was primed using random primers (30 ng). For subsequent PCR reaction, 10 ng of ethanol-precipitated cDNA was used as the template. The primer sequences are shown in Table 1. PCR was programmed for 28 cycles and the cycling conditions for PCR were as follows: 94°C for 3 min, annealing at 50°C for 30 s, and extension for 30 s at 72°C. Melting curve analysis was performed after the last cycle. All PCR reactions were run in triplicate. The mRNA expression levels of FHIT, P73 and IL-8 were calculated from the standard curve, and quantitative normalization in each sample was performed using GADPH gene expression as an internal control. Quantification was performed using the 2^-ΔΔCt^ method.

**Table 1:** Sequences of the primers used for amplification of FHIT, IL-8 and P73.

Amplified gene	Primer sequence	Amplification size (bp)
FHIT	Forward: 5’-TTG GGG CGC GGG TTT GGG TTT TTA CGC-3’	225
	Reverse: 5’-CGT AAA CGA CGC CGA CCC CAC TA-3’	
IL-8	Forward: 5'-ATG ACT TCC AAG GTG GCC GTG GCT-3'	293
	Reverse: 5'-TCT CCA GCC CTC TTC AAA AAC TTCT-3'	
P73	Forward: 5’-GAC GGA ATT CAC CAC CAT CCT-3’	389
	Reverse: 5’-CCA GGC TCT CTT TCA GCT TCA-3’	
GADPH	Forward: 5'-CAT CAT CTC TGC CCC CTC TG-3'	159
	Reverse: 5'-TCC ACG ATA CCA AAG TTG TC-3'	

### Statistical analysis

All statistical analyses were performed using SPSS 17.0 statistical software (Chicago, IL, USA). The differences in clinical characteristics including gender, smoking, drinking and histopathological change in the gastric mucosa were determined using Pearson’s *χ* ^2^ test and Fisher’s exact test. The differences in age, scores of protein expression, and levels of mRNA expression were determined using Kruskal-Wallis rank test. *P*≤0.05 was considered statistically significant.

## Results

### Demographics of patients

The numbers of patients in groups A, B, C and D were 9, 61, 6 and 56, respectively. As shown in Table 2, no significant differences were observed in the ration of males to females (*P*>0.05), median age (*P*>0.05), proportion of smoking (*P*>0.05), proportion of drinking (*P*>0.05) and proportion of different histopathological change in gastric mucosa (*P*>0.05) among the four groups.

**Table 2:** Clinical characteristics of patients in this study.

Terms	Group A (n = 9)	Group B (n = 61)	Group C (n = 6)	Group D (n = 56)	*P* value
Median age (years)	38	42	40.5	43.5	0.835
Gender (M/F)	5/4	40/21	4/2	32/24	0.716
Smoking	4	40	4	35	0.673
Drinking	6	55	5	49	0.268
Histopathological change of gastric mucosa					0.703
Non-atrophic gastritis	6	47	5	49	
Atrophic gastritis	2	10	1	5	
Intestinal metaplasia	1	4	0	2	

This is the Table 2 legend. Group A: patients with both *H*. *pylori* infection and family histories of gastric cancer. Group B: patients with *H*. *pylori* infection and without family histories of gastric cancer. Group C: patients without *H*. *pylori* infection and with family histories of gastric cancer. Group D: patients without both *H*. *pylori* infection and family histories of gastric cancer.

### Effect of *H*. *pylori* infection on expression of FHIT, IL-8 and P73 in gastric mucosa

**Fig 2:**
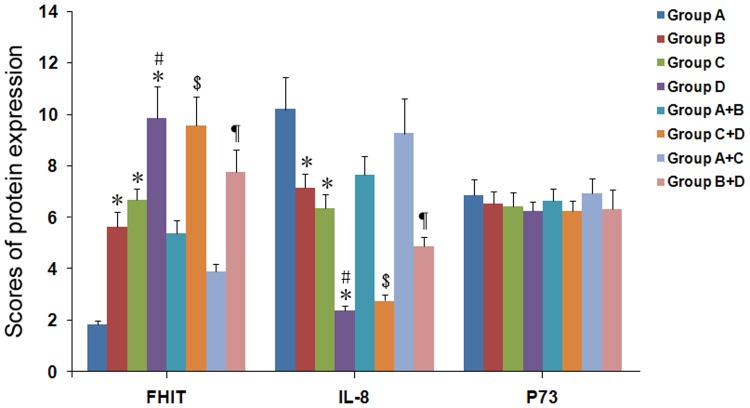
Effect of *H*. *pylori* infection and family histories of gastric cancer on protein expression of FHIT, IL-8 and P73 before eradication of *H*. *pylori*. Group A: patients with both *H*. *pylori* infection and family histories of gastric cancer. Group B: patients with *H*. *pylori* infection and without family histories of gastric cancer. Group C: patients without *H*. *pylori* infection and with family histories of gastric cancer. Group D: patients without both *H*. *pylori* infection and family histories of gastric cancer. Data were expressed as the mean ±standard deviation. Statistical differences were determined by Kruskal-Wallis rank test. Compared with Group A, * *P*<0.01; compared with Group B, # *P*<0.05; compared with Group A+B, $; compared with Group A+C, ¶ *P*<0.05.

**Fig 3:**
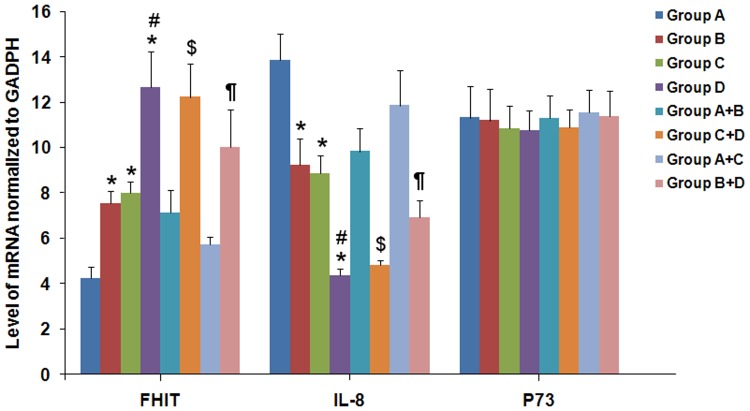
Effect of *H*. *pylori* infection and family histories of gastric cancer on mRNA expression of FHIT, IL-8 and P73 before eradication of *H*. *pylori*. Group A: patients with both *H*. *pylori* infection and family histories of gastric cancer. Group B: patients with *H*. *pylori* infection and without family histories of gastric cancer. Group C: patients without *H*. *pylori* infection and with family histories of gastric cancer. Group D: patients without both *H*. *pylori* infection and family histories of gastric cancer. Data were expressed as the mean±standard deviation. Statistical differences were determined by Kruskal-Wallis rank test. Compared with Group A, * *P*<0.01; compared with Group B, # *P*<0.05; compared with Group A+B, $; compared with Group A+C, ¶ *P*<0.05.

The protein and mRNA levels of FHIT were significantly lower in patients with *H*. *pylori* infection and without family histories of gastric cancer than those without *H*. *pylori* infection and family histories of gastric cancer (*P*<0.05; Figs 2 and 3), these levels were also significantly lower in patients with *H*. *pylori* infection when compared with those with no *H*. *pylori* infection (*P*<0.05; Figs 2 and 3).

In contrast, both the protein and mRNA levels of IL-8 were significantly elevated in patients with *H*. *pylori* infection and no family histories of gastric cancer than those without both *H*. *pylori* infection and family histories of gastric cancer (*P*<0.05; Figs 2 and 3), and they were also significantly higher in patients with *H*. *pylori* infection than those without *H*. *pylori* infection (*P*<0.05; Figs 2 and 3).

However, *H*. *pylori* infection had no major effect on the protein and mRNA expression levels of P73.

### Effect of family histories of gastric cancer on expression of FHIT, IL-8 and P73 in gastric mucosa

The protein and mRNA levels of FHIT were significantly lower in patients with family histories of gastric cancer and without *H*. *pylori* infection than those without *H*. *pylori* infection and family histories of gastric cancer (*P*<0.05; Figs 2 and 3), and were also significantly lower in patients with family histories of gastric cancer when compared with those without family histories of gastric cancer (*P*<0.05; Figs 2 and 3).

In opposition, the protein and mRNA levels of IL-8 were significantly higher in patients with family histories of gastric cancer and without *H*. *pylori* infection than those without both *H*. *pylori* infection and family histories of gastric cancer (*P*<0.05; Figs 2 and 3), and they were also significantly higher in patients with family histories of gastric cancer than those without family histories of gastric cancer (*P*<0.05; Figs 2 and 3).

However, family histories of gastric cancer had no major effect on the protein and mRNA expression levels of P73.

### Effects of eradication of *H*. *pylori* on expression of FHIT, IL-8 and P73

**Fig 4:**
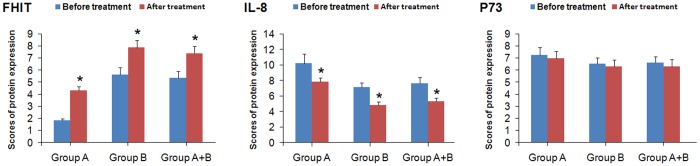
Effect of eradication of *H*. *pylori* on protein expression of FHIT, IL-8 and P73. Group A, patients with both *H*. *pylori* infection and family histories of gastric cancer. Group B, patients with *H*. *pylori* infection and without family histories of gastric cancer. Data were expressed as the mean±standard deviation. Statistical differences were determined by Wilcoxon rank-sum test. Compared with before treatment, * *P*<0.05.

**Fig 5:**
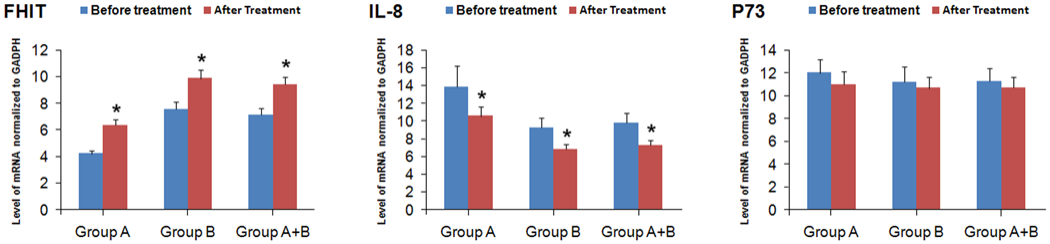
Effect of eradication of *H*. *pylori* on mRNA expression of FHIT, IL-8 and P73. A, patients with both *H*. *pylori* infection and family histories of gastric cancer. Group B, patients with *H*. *Pylori* infection and without family histories of gastric cancer. Data were expressed as the mean±standard deviation. Statistical differences were determined by Wilcoxon rank-sum test. Compared with before treatment, * *P*<0.05.

Both the protein and mRNA levels of FHIT were significantly higher after eradication of *H*. *pylori* than before eradication of *H*. *pylori* in patients with *H*. *pylori* infection and family histories of gastric cancer (*P*<0.05; Figs 4 and 5), in patients with *H*. *pylori* infection and without family histories of gastric cancer (*P*<0.05; Figs 4 and 5), or in patients with *H*. *pylori* infection (*P*<0.05; Figs 4 and 5).

By contrast, both the protein and mRNA levels of IL-8 were significantly lower after eradication of *H*. *pylori* than those before eradication of *H*. *pylori* in patients with *H*. *pylori* infection and family histories of gastric cancer (*P*<0.05; Figs 4 and 5), in patients with *H*. *pylori* infection and without family histories of gastric cancer (*P*<0.05; Figs 4 and 5), or in patients with *H*. *pylori* infection (*P*<0.05; Figs 4 and 5).

However, eradication of *H*. *pylori* had no major effect on the protein and mRNA expression levels of P73.

## Discussion

In this study, we show that *H*. *pylori* infection or family histories of gastric cancer may correlate with a down-regulation of the FHIT expression and up-regulation IL-8 expression levels in the gastric mucosa of patients with functional dyspepsia. Eradication of *H*. *pylori* may lead to up-regulation FHIT expression and down-regulation IL-8 expression in first-degree relatives of gastric cancer. However, *H*. *pylori* infection or family histories of gastric cancer had no significant effect on P73 expression.

FHIT has been proven to regulate cell cycle, cell apoptosis and cell proliferation of tumor cells [33–35]. Ishii et al showed that the transduction of the FHIT gene into seven esophageal cancer cell lines induced caspase-dependent apoptosis in two cell lines which expressed no or very little FHIT [36]. Similarly, the same authors also reported that viral vector-mediated FHIT gene transfer to FHIT-deficient mice not only prevented but also reversed carcinogen-induced tumor development in vivo [37]. Stec-Michalska et al reported that the FHIT mRNA expression in patients with dyspepsia and family histories of gastric cancer is by 32% lower than the levels measured with patients without family histories of gastric cancer [20]. Although in 2009, Stec-Michalska et al further increased sample size and improved the experimental methods and the results were similar [21]. Our data also show that FHIT is significantly decreased in first-degree relatives of gastric cancer compared with those without such relatives. Therefore, the FHIT gene may be one of the targets of the genetic susceptibility to gastric cancer in patients with family histories of gastric cancer, especially first-degree relatives of gastric cancer.

Gastric cancer is closely related to environmental carcinogens, especially *H*. *pylori* infection [2–4]. The decrease or missing expression of the FHIT protein is correlates well with the occurrence of *H*. *pylori* infection [21]. Skopelitou et al showed that the rates of reduction or loss of FHIT protein expression in *H*. *pylori*-related gastritis, chronic gastritis associated with mild and severe dysplasia, and gastric cancer were 79%, 76% and 56%, respectively [38]. Our results indicated that FHIT expression is significantly higher in the first-degree relatives of gastric cancer after eradication of *H*. *pylori*. Therefore, eradication of *H*. *pylori* is highly important for populations with family histories of gastric cancer, especially first-degree relatives of gastric cancer.

IL-8 can regulate neovascularization, and then promote the growth and spread of human gastric carcinoma [39]. Yamaoka et al found that the expression of IL-8 in gastric cancer tissues was 10 times higher than that in normal tissue, and was twice the amount in advanced gastric cancer tissues than in early cancer tissues [40]. The relationship between host gene polymorphism and gastric cancer has recently become a research hotspot. IL-8 gene polymorphisms include IL-8-251 A/T, -396 T/G and -781 C/T [41]. In particular, the IL-8-251 A/A genotype significantly increased the risk of cardiac cancer, especially in patients with definite family histories of cancer of the gastrointestinal tract and/or patients with *H*. *pylori* infection, whereas the IL- 8–251 A allele is associated with over-expression of IL-8 [42]. Our results demonstrated that IL-8 expression was significantly higher in patients with family histories of gastric cancer. Although, the data suggest that family histories of gastric cancer may be associated with the inflammatory process in the gastric mucosa through the over-expression of IL-8.

Gastric cancer development is a complex process involving the interaction of genetic and environmental factors. *H*. *pylori* infection is considered an important factor in the cause of gastric cancer, and IL-8 levels are significantly higher in gastric mucosa infected with *H*. *pylori* infection [43, 44]. Our results also showed that IL-8 expression significantly decreased after eradication of *H*. *pylori*. Persistent *H*. *pylori* infection induces IL-8 expression, damages the gastric mucosa, and eventually leads to the carcinogenesis and development of gastric cancer [1]. Thus, IL-8 expression is significant for populations with family histories of gastric cancer to eradicate *H*. *pylori*.

The location of the P73 gene and structural similarity to P53 suggest that it may be a tumor suppressor gene. However, P73 is expressed at low levels in all normal tissues and is not induced by UV irradiation or actinomycin D, which is known to elevate P53 expression [45, 46]. In addition, over-expression of P73, different from P53, occurs in breast cancer, gastric cancer, colon cancer, lung cancer [47]. Thus, P73 has been questioned as a tumor suppressor gene. Carrasco et al, used the tissue chip technique to detect non-tumor adjacent gastric mucosa in 91 patients with early gastric cancer and gastric mucosa in 148 patients with chronic gastritis, and found that the positive rate of P73 expression in non-tumor adjacent gastric mucosa (50.5%) was significantly higher than that (10.8%) in chronic gastritis (*P*<0.01) [27]. However, Ge et al reported that the family histories of upper gastrointestinal cancer increased the risk of developing esophageal squamous cell carcinoma, but the overall distribution of the P73 genotype, allelotype and haplotype in cancer patients and controls were not significantly different [48]. Moreover, specific mutations of P73 were not identified in gastric carcinomas and unrelated with the prognosis of patients with gastric carcinomas [49]. Our results showed that *H*. *pylori* infection or family histories of gastric cancer had no major effect on the P73 expression in gastric mucosa of patients with functional dyspepsia. Kondo et al reported that accumulation of aberrant CpG hypermethylation by *H*. *pylori* infection promoted development and progression of gastric mucosa-associated lymphoid tissue (MALT) lymphoma, and no differences were observed in the mRNA level of P73 in the gastric mucosa of patients with *H*. *pylori*-dependent and-independent gastric MALT lymphoma; thus, the mRNA level of P73 did not significantly change before and after eradication of *H*. *pylori* [50]. Furthermore, Kang et al suggest that p73 might not be a target of genetic alteration in gastric carcinogenesis and over-expression of p73 may be triggered by physiological stresses accompanied with outgrowth of tumors, such ashypoxia or nutrient deprivation [51]. Hence, P73 may be not an independent risk factor for the genesis and development of gastric cancer.

In summary, FHIT and IL-8 may constitute targets of the genetic susceptibility to gastric cancer in patients with family histories of gastric cancer, especially first-degree relatives of gastric cancer. Down-regulation of FHIT and up-regulation of IL-8 may be involved in the pathogenesis of *H*. *pylori* infection.
